# Intelligence Test Scores Before and After Alcohol‐Related Disorders—A Longitudinal Study of Danish Male Conscripts

**DOI:** 10.1111/acer.14174

**Published:** 2019-08-24

**Authors:** Marie Grønkjær, Trine Flensborg‐Madsen, Merete Osler, Holger Jelling Sørensen, Ulrik Becker, Erik Lykke Mortensen

**Affiliations:** ^1^ Department of Public Health University of Copenhagen Copenhagen Denmark; ^2^ Center for Healthy Aging University of Copenhagen Copenhagen Denmark; ^3^ Center for Clinical Research and Disease Prevention Bispebjerg and Frederiksberg Hospital Copenhagen Denmark; ^4^ Mental Health Centre Copenhagen Copenhagen University Hospital Gentofte Gentofte Denmark; ^5^ National Institute of Public Health University of Southern Denmark Copenhagen Denmark; ^6^ Gastrounit Medical Division Copenhagen University Hospital Hvidovre Hvidovre Denmark

**Keywords:** Alcohol‐Related Disorders, Intelligence, Intelligence Quotient, Intellectual Changes, Longitudinal Study

## Abstract

**Background:**

Existing studies on intellectual consequences of alcohol‐related disorders are primarily cross‐sectional and compare intelligence test scores of individuals with and without alcohol‐related disorders, hence mixing the influence of alcohol‐related disorders and predisposing factors such as premorbid intelligence. In this large‐scale study, the primary aim was to estimate associations of alcohol‐related disorders with changes in intelligence test scores from early adulthood to late midlife.

**Methods:**

Data were drawn from a follow‐up study on middle‐aged men, which included a re‐examination of the same intelligence test as completed in young adulthood at military conscription (total analytic sample = 2,499). Alcohol‐related hospital diagnoses were obtained from national health registries, whereas treatment for alcohol problems was self‐reported at follow‐up. The analyses included adjustment for year of birth, retest interval, baseline intelligence quotient (IQ) score, education, smoking, alcohol consumption, and psychiatric and somatic comorbidity.

**Results:**

Individuals with alcohol‐related hospital diagnoses (8%) had a significantly lower baseline IQ score (95.0 vs. 100.5, *p* < 0.001) and a larger decline in IQ scores from baseline to follow‐up (−8.5 vs. −4.8, *p* < 0.001) than individuals without such diagnoses. The larger decline in IQ scores with alcohol‐related hospital diagnoses remained statistically significant after adjustment for all the covariates. Similar results were revealed when IQ scores before and after self‐reported treatment for alcohol problems (10%) were examined.

**Conclusions:**

Individuals with alcohol‐related disorders have a lower intelligence test score both in young adulthood and in late midlife, and these disorders, moreover, seem to be associated with more age‐related decline in intelligence test scores. Thus, low mean intellectual ability observed in individuals with alcohol‐related disorders is probably a result of both lower premorbid intelligence and more intellectual decline.

## Graphical abstract

In a large male study sample, lower intelligence test scores in young adulthood were observed in men with alcohol‐related disorders. Moreover, men with alcohol‐related disorders had a larger decline in intelligence test scores from young adulthood to late midlife than men without these disorders. Thus, lower intellectual ability in individuals with alcohol‐related disorders observed in clinical studies is probably a result of both lower premorbid intelligence and more age‐related intellectual decline in this group compared with individuals without alcohol‐related disorders.

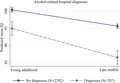

Long‐term consequences of heavy alcohol consumption and alcohol‐related disorders on intellectual aging are of particular interest with the aging population. Cross‐sectional studies of clinical samples comparing individuals with alcohol dependence and controls without alcohol dependence have observed lower cognitive performance on several tests, including tests of global cognitive function, speed of processing, and verbal and nonverbal cognitive ability (Kist et al., 2014; Kopera et al., 2012; Schottenbauer et al., 2007; Woods et al., 2016). Likewise, more cognitive impairments have been observed in individuals with alcoholic liver cirrhosis than in individuals with viral liver cirrhosis (Lee et al., 2016). However, cross‐sectional studies cannot disclose whether lower intellectual ability in individuals with alcohol‐related disorders is observed because low intelligence is a risk factor for alcohol‐related disorders or because low intelligence is a consequence of alcohol‐related disorders (Schottenbauer et al., 2007). Several studies have indicated that low intelligence is a risk factor for alcohol use disorders later in life (Mortensen et al., 2005; Osler et al., 2006; Sjölund et al., 2015). In contrast, studies on long‐term consequences of alcohol‐related disorders on intelligence are lacking, although the acute effects of alcohol on intellectual performance have long been documented (Dry et al., 2012; Peterson et al., 1990). Using magnetic resonance imaging (MRI), studies have shown shrinkage of the frontal cortex and underlying white matter in individuals with alcohol‐related disorders (Rosenbloom and Pfefferbaum, 2008), that is, damages in structures of the brain that are vital for intellectual functions. However, whether these damages are caused by direct effects of alcohol on the brain or indirect effects of other factors such as lifestyle and comorbidity still needs clarification. The importance of comorbidity is emphasized by the high prevalence of psychiatric and somatic comorbidity in individuals with alcohol‐related disorders (Flensborg‐Madsen et al., 2009; Holst et al., 2017). Clinical neuropsychological studies as well as MRI studies have indicated structural and functional improvement with long‐term abstinence (Stavro et al., 2013; Sullivan, 2017), suggesting that some of the intellectual impairment caused by alcohol is acute and does not pose permanent changes. In these studies, intellectual functions are assumed to be normalized if they are comparable to the functions of normal controls; however, without knowledge of intellectual performance before the alcohol use disorder was initiated, the *individual* premorbid intelligence is not known. Therefore, studies investigating the association of alcohol‐related disorders with long‐term intellectual changes that also account for premorbid intelligence and comorbidity are needed. Results of such studies can provide a better understanding of the long‐term consequences of heavy alcohol consumption and accompanying disorders and may help to prevent or postpone age‐related intellectual decline.

In a recently established follow‐up study, the Lifestyle and Cognition Follow‐up study 2015 (LiKO‐15), participants completed a comprehensive questionnaire in late midlife along with the same intelligence test as they did at the military draft board examination in early adulthood. Consequently, data from LiKO‐15 provide a unique opportunity to thoroughly investigate the associations between alcohol‐related disorders and intelligence while addressing many of the drawbacks of previous studies. A crucial element is that intelligence was assessed before and after initiation of alcohol‐related disorders. The primary aim was to estimate associations of alcohol‐related disorders with changes in intelligence test scores from early adulthood to late midlife. To highlight the importance of including premorbid intelligence test scores, a secondary aim was to estimate associations of intelligence test scores in young adulthood with alcohol‐related disorders in the same study sample.

## Materials and Methods

### Study Sample

The study utilized data from the Lifestyle and Cognition Follow‐up study 2015 (LiKO‐15) and national health registries. The LiKO‐15 is a follow‐up study of middle‐aged men (born in 1950 to 1961; mean age = 61.6 [SD = 3.3]) from 2 existing databases: a psychiatric database established by Urfer‐Parnas and colleagues (2010) and the Danish Conscription Database (Christensen et al., 2015), both including information on intelligence test scores from draft board examinations in early adulthood (1968 to 1989). At the follow‐up examination in late midlife (2015 to 2017), LiKO‐15 participants were re‐assessed using the same intelligence test and answered a comprehensive questionnaire on socio‐demographic factors, lifestyle and health. In total, 19,888 men were invited to participate in the follow‐up of whom 2,611 men (13%) agreed to participate. Results of a participation analysis—presented elsewhere (Grønkjær et al., 2019b)—have indicated that participants in LiKO‐15 constitute a rather selected group of men with higher education and intelligence test scores in young adulthood and less morbidity than nonparticipants. As the main aims of LiKO‐15 were to investigate associations between psychiatric disorders and age‐related intellectual changes, a large proportion of the invited men had been registered with psychiatric hospital diagnoses (46%), including alcohol use disorders (15%). Data on 89 participants were lost due to technical problems in the military's computer systems, 2 participants had alcohol‐related diagnoses before baseline, and 21 participants were excluded as they had missing values on 1 or more of the included variables; hence, 2,499 men were included in the analyses. By use of the unique personal identification number, which is assigned to all Danish residents (Pedersen, 2011), data were linked to the Danish National Patient Registry, containing information on all inpatient admissions to Danish hospitals since 1977 and outpatient admissions since 1995 (Lynge et al., 2011). Moreover, information on all inpatient admissions to Danish psychiatric hospital departments since 1969 and outpatient admissions since 1995 was obtained from the Danish Psychiatric Central Research Register (Mors et al., 2011).

### Alcohol‐Related Disorders

Alcohol‐related disorders were assessed using alcohol‐related hospital diagnoses—including both psychiatric and somatic diagnoses—and self‐reported treatment for alcohol problems. The somatic alcohol‐related hospital diagnoses were included as they capture individuals who have had an alcohol‐related disorder (i.e., at least harmful use of alcohol) but who have not necessarily been in contact with the psychiatric hospital departments. The decline in intelligence test scores with both alcohol‐related hospital diagnoses and self‐reported treatment for alcohol problems was investigated as they have different strengths and limitations, hence enabling comparison of the results using different operationalizations of alcohol‐related disorders. Information on psychiatric alcohol‐related hospital diagnoses from psychiatric wards was acquired from the Danish Psychiatric Central Research Register and the Danish National Patient Registry using the following diagnoses: ICD‐8 (291.09 to 291.99, 303.09 to 303.99) and ICD‐10 (F10.1 to F10.9). Somatic alcohol‐related hospital diagnoses were obtained from the Danish National Patient Registry including the following diagnoses: ICD‐8 (571.09, 571.10) and ICD‐10 (E24.4, G31.2, G62.1, G72.1, I42.6, K29.2, K70, K85.2, K86.0). At the follow‐up examination, participants self‐reported whether they had ever been treated for alcohol problems with the response categories: yes, several times; yes, once; and no (in analyses dichotomized into yes/no).

### Intelligence

Intelligence was assessed using test scores on the global intelligence test Børge Priens Prøve (BPP), which is highly correlated with the full‐scale intelligence quotient (IQ) of the Wechsler Adult Intelligence Scale (Mortensen et al., 1989). The BPP is used by the Danish military draft authorities and comprises 78 items within subtests of letter matrices, number series, verbal analogies, and geometric figures (Teasdale, 2009). Participants for the present paper were first assessed using a paper‐and‐pencil version of BPP in young adulthood (baseline; mean age = 20.3 years) and re‐assessed using a computerized version of BPP in late midlife (follow‐up; mean age = 61.6 years). The total number of correct answered items on BPP was available from both baseline and follow‐up. As only the total BPP score was archived by the draft board authorities, BPP subtest scores were only available from follow‐up examinations. Thus, changes in subtest scores could not be investigated, but a previous study also indicated that BPP is probably more suitable to investigate overall changes than changes in specific subtests as a factor analysis found that a 1‐factor model fits the BPP subtests (Grønkjær et al., 2019a). BPP test scores were converted to an IQ scale with a sample mean of 100 and a standard deviation of 15 at baseline, and changes in IQ scores were used to assess age‐related intellectual changes.

### Covariates

At the follow‐up examination, participants retrospectively reported information on school and vocational education, lifetime tobacco smoking, and lifetime alcohol consumption. Based on this information, we calculated the number of years of education using nominal lengths of study times, and pack‐years of smoking using the following tobacco equivalents: 1 cigarette = 1 unit, 1 cigarillo = 3 units, 1 cigar = 5 units, and 1 pipe = 3 units (Christensen et al., 2008). In addition, the average weekly units of alcohol and years with weekly extreme binge drinking (≥10 units of alcohol on the same occasion) in adult life were calculated. As described in detail elsewhere (Grønkjær et al., 2019b), adult life was defined as 26 years of age and onwards and the Danish units of alcohol corresponding to 12 g of pure alcohol were applied in these calculations.

Using information on hospital diagnoses from psychiatric departments obtained through the Danish Psychiatric Central Research Register and the Danish National Patient Registry, the following psychiatric disorder variables were constructed: Other substance use disorders (ICD‐8: 304 and ICD‐10: F11 to F19); Schizophrenia (ICD‐8: 295 and ICD‐10: F20); Mood disorders (ICD‐8: 296 and ICD‐10: F30 to F39); and Other psychiatric disorders (ICD‐8: 290 to 302 and ICD‐10: F11 to F99 [excluding the diagnostic codes specified in the other groups]). Moreover, information from the Danish National Patient Registry was used to calculate the Charlson Comorbidity Index to assess somatic comorbidity (Charlson et al., 1987). The index includes 19 comorbidities that are weighted based on their potential influence on mortality using the following weights: 1 (myocardial infarction; congestive heart failure; peripheral vascular disease; cerebrovascular disease; dementia; chronic pulmonary disease; connective tissue disease; peptic ulcer disease; mild liver disease; and diabetes without end‐organ damage); 2 (diabetes with end‐organ damage; hemiplegia; moderate‐to‐severe renal disease; tumor without metastasis; leukemia; and lymphoma); 3 (moderate‐to‐severe liver disease); or 6 (metastatic solid tumor and AIDS). In addition, year of birth was included as a covariate in the analyses to adjust for different ages at follow‐up and to adjust for generation differences in alcohol‐related disorders and intelligence.

### Statistical Methods

Study sample characteristics by alcohol‐related hospital diagnoses and self‐reported treatment for alcohol problems were analyzed for group differences using chi‐square tests for categorical variables and independent‐sample *t*‐tests for continuous variables (Table 1). Associations of IQ scores with alcohol‐related hospital diagnoses and self‐reported treatment for alcohol problems were analyzed using independent‐sample *t*‐tests of group differences in baseline IQ scores, follow‐up IQ scores, and changes in IQ scores (Table 2). Moreover, dependent‐sample *t*‐test was used to analyze within‐group changes in IQ scores from baseline to follow‐up. Linear regression analyses of changes in IQ scores with alcohol‐related hospital diagnoses and self‐reported treatment for alcohol problems were conducted adjusted for (i). year of birth, retest interval length, baseline IQ score, years of education, and pack‐years of smoking (Model 1); (ii) Model 1 + psychiatric and somatic comorbidity; (iii) Model 1 + average weekly units of alcohol consumed in adult life; and (iv) Model 1 + years with weekly extreme binge drinking (≥10 units the on same occasion) in adult life (Table 3). The covariates included in the Model 1‐adjusted analyses were selected based on the assumption that these factors are likely to influence both alcohol‐related disorders and age‐related intellectual decline. The covariates included in Models 2 to 4 can be interpreted as either confounders or mediators of the association; hence, the results of these analyses were presented separately. Moreover, variance in IQ score changes uniquely explained by alcohol‐related hospital diagnoses and self‐reported treatment for alcohol problems (increment in *R* ^2^) was estimated and presented. Finally, linear regression analyses of changes in IQ scores with alcohol‐related hospital diagnoses and self‐reported treatment for alcohol problems were conducted on a subsample of participants excluding men with alcohol‐related diagnoses the past year (*N* = 33), in analyses only including currently abstinent cases (i.e., excluding cases with alcohol consumption the past year), and in analyses including adjustment for heavy alcohol consumption the past year in terms of more than 21 units of alcohol per week or weekly extreme binge drinking (Table 4; Model 1‐adjusted estimates presented). The linear regression model assumptions were evaluated, but no violations were observed. All analyses were performed using Stata 14.

**Table 1:** Characteristics of the Study Sample of 2,499 Danish Men by Alcohol‐Related Hospital Diagnoses and Self‐Reported Treatment for Alcohol Problems

	Alcohol‐related hospital diagnoses		Self‐reported treatment for alcohol problems	
	Yes (*n* = 207)	No (*n* = 2,292)^a^	Yes (*n* = 256)	No (*n* = 2,243)^a^
Age at draft board examination (mean, SD)	20.1 (1.9)	20.3 (2.1)	20.2 (1.9)	20.3 (2.1)
Age at follow‐up examination (mean, SD)	62.2 (3.1)	61.6 (3.3)**	62.2 (3.0)	61.6 (3.3)**
Retest interval (mean, SD)	42.1 (3.5)	41.3 (3.3)***	42.1 (3.4)	41.3 (3.3)***
Years of education (mean, SD)	12.7 (2.7)	13.7 (2.5)***	12.8 (2.7)	13.7 (2.5) ***
Pack‐years of smoking (mean, SD)	38.1 (30.8)	17.8 (25.9)***	37.6 (30.1)	17.5 (25.7) ***
Adult‐life weekly units of alcohol (mean, SD)	26.7 (27.9)	11.1 (9.8)***	28.1 (27.2)	10.6 (8.7) ***
Years with weekly extreme binge drinking (mean, SD)^b^	16.2 (14.5)	2.8 (8.0)***	18.0 (13.5)	2.3 (7.3)***
Charlson Comorbidity Index score (mean, SD)^c^	1.7 (2.2)	0.8 (1.5)***	1.6 (2.2)	0.8 (1.5)***
Other substance use disorders (*N*, %)^d^	4 (1.9)	21 (0.9)	4 (1.6)	21 (0.9)
Schizophrenia (*N*, %)^d^	10 (4.8)	41 (1.8)**	12 (4.7)	39 (1.7)**
Mood disorders (*N*, %)^d^	73 (35.3)	184 (8.0)***	69 (27.0)	188 (8.4)***
Other psychiatric disorders (*N*, %)^d^	122 (58.9)	557 (24.3)***	136 (53.1)	543 (24.2)***

a Chi‐square test for categorical variable and independent‐sample *t*‐tests for continuous variables.

b Defined as consuming 10 units of alcohol or more on the same occasion.

c Calculated based on information from the Danish National Patient Registry.

d Based on diagnoses from Danish psychiatric hospital wards.

**p ≤ 0.01, ***p ≤ 0.001.

One unit of alcohol = 12 g of pure alcohol.

**Table 2:** IQ scores Before and After Alcohol‐Related Hospital Diagnoses and Self‐Reported Treatment for Alcohol Problems in 2,499 Danish Men

Groups and differences	Mean baseline IQ score (SD/SE) (min; max)	Mean follow‐up IQ score (SD/SE) (min; max)	Mean change in IQ (SD/SE)
Alcohol‐related hospital diagnoses			
Yes (*N* = 207)	95.0 (17.2) (44; 133)	86.4 (16.9) (38; 126)	−8.5 (11.6)***
No (*N* = 2,292)	100.5 (14.7) (40; 136)	95.7 (14.2) (38; 138)	−4.8 (9.0)***
Difference_crude_	−5.5 (1.1)***	−9.2 (1.0)***	−3.8 (0.7)***
Self‐reported treatment for alcohol problems			
Yes (*N* = 256)	96.2 (16.4) (44; 133)	88.2 (16.6) (38; 127)	−8.0 (10.6)***
No (*N* = 2,243)	100.4 (14.8) (40; 136)	95.7 (14.2) (38; 138)	−4.8 (9.1)***
Difference_crude_	−4.2 (1.0)***	−7.4 (1.0)***	−3.2 (0.6)***

IQ = intelligence quotient calculated using Børge Priens Prøve test scores.

***p ≤ 0.001, independent‐sample *t*‐test of group mean comparisons and dependent‐sample *t*‐test of within‐group mean changes in IQ scores.

**Table 3:** Adjusted Estimates of Changes in IQ Scores With Alcohol‐Related Hospital Diagnoses and Self‐Reported Treatment for Alcohol Problems in 2,499 Danish Men

	Mean group difference in IQ changes (95% CI)	*p*‐Value	Increment in R^2^ by the alcohol‐related disorder variable, %
Alcohol‐related hospital diagnoses (*N*_cases_ = 207)			
Model 1‐adjusted estimates	−4.8 (−6.1; −3.6)	<0.001	2.0
Model 2 (Model 1 + psychiatric and somatic comorbidity)	−3.6 (−4.8; −2.3)	<0.001	1.0
Model 3 (Model 1 + average units of alcohol/wk in adult life)	−4.7 (−6.0; −3.4)	<0.001	1.7
Model 4 (Model 1 + years with weekly extreme binge drinking in adult life)	−3.8 (−5.1; −2.5)	<0.001	1.1
Self‐reported treatment for alcohol problems (*N*_cases_ = 256)			
Model 1‐adjusted estimates	−3.9 (−5.1; −2.8)	<0.001	1.5
Model 2 (Model 1 + psychiatric and somatic comorbidity)	−2.9 (−4.0; −1.8)	<0.001	0.8
Model 3 (Model 1 + average units of alcohol/wk in adult life)	−3.9 (−5.1; −2.6)	<0.001	1.3
Model 4 (Model 1 + years with weekly extreme binge drinking in adult life)	−2.7 (−4.0; −1.4)	<0.001	0.6

IQ = intelligence quotient calculated using Børge Priens Prøve test scores, Model 1: adjusted for year of birth, retest interval length, baseline IQ score, years of education, and pack‐years of smoking.

One unit of alcohol = 12 g of pure alcohol. Extreme binge drinking defined as consuming 10 units of alcohol or more on the same occasion.

**Table 4:** Model 1‐Adjusted Estimates of Changes in IQ Scores With Alcohol‐Related Hospital Diagnoses and Self‐Reported Treatment for Alcohol Problems in 2,499 Danish Men When Excluding Individuals With Recent Alcohol‐Related Hospital Diagnoses, Only Including Currently Abstinent Cases and Adjusting for Heavy Alcohol Consumption the Past Year

	Cases/noncases	Mean group difference in IQ changes (95% CI)	*p*‐Value
Excluding men with alcohol‐related diagnoses the past year (*N* = 33)			
Alcohol‐related hospital diagnosis	174/2,292	−3.8 (−5.1; −2.4)	<0.001
Self‐reported treatment for alcohol problems	236/2,230	−3.6 (−4.8; −2.5)	<0.001
Only including currently abstinent cases (no consumption past year)			
Alcohol‐related hospital diagnosis	103/2,292	−3.5 (−5.1; −1.8)	<0.001
Self‐reported treatment for alcohol problems	138/2,243	−3.0 (−4.4; −1.5)	<0.001
Adjusted for >21 units/wk past year			
Alcohol‐related hospital diagnosis	207/2,292	−4.8 (−6.1; −3.6)	<0.001
Self‐reported treatment for alcohol problems	256/2,243	−3.9 (−5.0; −2.8)	<0.001
Adjusted for weekly extreme binge drinking past year			
Alcohol‐related hospital diagnosis	207/2,292	−4.6 (−5.8; −3.3)	<0.001
Self‐reported treatment for alcohol problems	256/2,243	−3.6 (−4.8; −2.5)	<0.001

IQ = intelligence quotient calculated using Børge Priens Prøve test scores, Model 1: adjusted for year of birth, retest interval length, baseline IQ score, years of education, and pack‐years of smoking.

One unit of alcohol = 12 g of pure alcohol. Extreme binge drinking defined as consuming 10 units of alcohol or more on the same occasion.

## Results

### Study Sample Characteristics

The study sample consisted of 2,499 men with a total of 366 psychiatric alcohol‐related hospital diagnoses and 49 somatic alcohol‐related hospital diagnoses (Table S1). These diagnoses were distributed on 207 men (8%) who had either only psychiatric alcohol‐related hospital diagnoses (*N* = 167), only somatic alcohol‐related hospital diagnoses (*N* = 13), or both (*N* = 27) (note that the total number of diagnoses adds up to more than the total number of individuals with alcohol‐related hospital diagnoses because some individuals have been registered with more than 1 alcohol‐related hospital diagnosis). The mean age at first registration with psychiatric alcohol‐related hospital diagnosis was 45.4 years (range: 21 to 66, SD = 10.9), and the mean number of registrations was 6.1 (SD = 13.1). In relation to somatic alcohol‐related hospital diagnoses, the mean age at first registration was 50.0 years (range: 32 to 67, SD = 8.1) and the mean number of registrations was 4.5 (SD = 5.7). At the follow‐up examination, 256 participants (10.2%) self‐reported that they had been treated for alcohol problems either once (5.7%) or several times (4.6%). The vast majority of participants registered with alcohol‐related hospital diagnoses also self‐reported treatment for alcohol problems (73.4%) in the questionnaire. Nevertheless, differences between the 2 alcohol‐related disorder operationalizations were also present; 55 men had alcohol‐related hospital diagnoses but did not self‐report treatment for alcohol problems, while 104 men reported treatment for alcohol problems but did not have an alcohol‐related hospital diagnosis.

Study sample characteristics by alcohol‐related hospital diagnoses and self‐reported treatment for alcohol problems are shown in Table 1. Compared with individuals with no alcohol‐related hospital diagnoses, individuals with alcohol‐related hospital diagnoses had significantly less years of education, more pack‐years of smoking, and more excessive alcohol consumption, including both average weekly consumption and extreme binge drinking. Moreover, individuals with alcohol‐related hospital diagnoses displayed more somatic as well as psychiatric comorbidity than individuals without such diagnoses. Similar patterns were observed for self‐reported treatment for alcohol problems.

Of the 78 items in BPP, participants had a mean BPP score of 46.2 (SD = 9.8) at draft board examination (baseline; mean age = 20.3 years). The raw BPP scores were converted to a baseline mean IQ score of 100 (SD = 15). At the follow‐up examination (mean age = 61.6 years), participants had a significantly lower mean IQ score of 94.9 (SD = 14.6; dependent‐sample *t*‐test: *p* < 0.001); hence, during the mean retest interval of 41.3 years (range: 27 to 48, SD = 3.4), participants had an average decline of −5.1 (SD = 9.3) IQ points.

### Intelligence Test Scores Before Alcohol‐Related Disorders

Participants with alcohol‐related hospital diagnoses had a significantly lower mean baseline IQ score than individuals without such diagnoses (Crude baseline IQ difference = −5.5 [SE = 1.1]; *p* < 0.001) (Table 2). When IQ scores were stratified by self‐reported treatment for alcohol problems, the results observed were largely similar to those observed with alcohol‐related hospital diagnoses; thus, participants with self‐reported treatment for alcohol problems had a lower baseline IQ score than individuals with no self‐reported treatment (Crude baseline IQ difference = −4.2 [SE = 1.0]; *p* < 0.001).

### Alcohol‐Related Disorders and Changes in Intelligence Test Scores

Participants with alcohol‐related hospital diagnoses had significantly larger decline in IQ scores from baseline to follow‐up than individuals without these diagnoses (Crude difference in IQ changes = −3.8 [SE = 0.7]; *p* < 0.001) (Table 2). The estimated decline in IQ scores with alcohol‐related hospital diagnoses became even larger after adjustment for primary covariates (Model 1‐adjusted difference in IQ changes = −4.8 [95% CI: −6.1; −3.6]; *p* < 0.001) (Table 3), which was primarily due to adjustment for baseline IQ scores and years of education (both of which were associated with larger decline in IQ scores). In the Model 1‐adjusted analyses, alcohol‐related diagnoses uniquely explained 2% of the variance in changes in IQ scores. The estimated decline in IQ scores with alcohol‐related hospital diagnoses was attenuated but remained statistically significant after further adjustment for comorbidity and adult‐life alcohol consumption (Models 2 to 4 in Table 3). When analyses were conducted comparing individuals with *somatic* alcohol‐related hospital diagnoses (*N* = 40) and individuals without these diagnoses, an even larger decline in IQ scores from baseline to follow‐up was observed (Model 1‐adjusted difference in IQ changes = −7.6 [95% CI: −10.2; −4.9]; *p* < 0.001) (Table S2). In addition, participants with self‐reported treatment for alcohol problems had larger decline in IQ scores from baseline to follow‐up than participants not reporting treatment for alcohol problems (Model 1‐adjusted difference in IQ changes = −3.9 [95% CI: −5.1; −2.8]; *p* < 0.001) and self‐reported treatment for alcohol problems uniquely explained 1.5% of the variance in changes in IQ scores (Table 3).

The estimated changes in IQ scores with alcohol‐related hospital diagnoses and self‐reported treatment for alcohol problems were marginally attenuated by excluding individuals with alcohol‐related hospital diagnoses within the past year (e.g., alcohol‐related hospital diagnoses excluding past‐year diagnoses: Difference in IQ changes = −3.8 [95% CI: −5.1; −2.4]; *p* < 0.001) (Table 4). Moreover, in analyses only including currently abstinent cases, the group differences in changes in IQ scores with alcohol‐related hospital diagnoses and self‐reported treatment for alcohol problems were smaller yet remained statistically significant (e.g., alcohol‐related hospital diagnoses including only past‐year abstinent cases: Difference in IQ changes = −3.5 [95% CI: −5.1; −1.8]; *p* < 0.001) (Table 4). Finally, the estimated changes in IQ scores with alcohol‐related hospital diagnoses and self‐reported treatment for alcohol problems were largely unaffected by adjustment for past‐year weekly consumption of 21 units of alcohol or more (e.g., alcohol‐related hospital diagnoses adjusted for past‐year consumption >21 units: Difference in IQ changes = −4.8 [95% CI: −6.1; −3.6]; *p* < 0.001) and weekly extreme binge drinking (≥10 units on the same occasion) (e.g., alcohol‐related hospital diagnoses adjusted for past‐year weekly extreme binge drinking: Difference in IQ changes = −4.6 [95% CI: −5.8; −3.3]; *p* < 0.001).

## Discussion

### Main Findings

In this longitudinal study using 2 different assessment methods of alcohol‐related disorders in combination with intelligence tested in early adulthood and late midlife, lower intelligence test scores at baseline were observed in individuals with alcohol‐related hospital diagnoses and self‐reported treatment for alcohol problems. Moreover, individuals with alcohol‐related disorders had a larger decline in intelligence test scores than individuals without such disorders regardless of the assessment method of alcohol‐related disorders. Thus, largely similar results were observed when alcohol‐related disorders were assessed using alcohol‐related hospital diagnoses and self‐reported treatment for alcohol problems, while the largest group difference in changes in intelligence test scores was observed for the *somatic* alcohol‐related hospital diagnoses. The differences in changes in intelligence test scores associated with alcohol‐related disorders could not be explained by educational level, baseline IQ score, pack‐years of smoking, adult‐life alcohol consumption, or psychiatric and somatic comorbidity. Moreover, the results of changes in intelligence test scores with alcohol‐related disorders were robust in relation to exclusion of individuals with alcohol‐related hospital diagnoses within the past year and only inclusion of currently abstinent cases and adjustment for heavy alcohol consumption within the past year.

### Intelligence Test Scores Before Alcohol‐Related Disorders

The association between low baseline intelligence test scores and alcohol‐related disorders observed in our male study sample is in accordance with findings from previous longitudinal studies on male conscripts (Mortensen et al., 2005; Sjölund et al., 2012) as well as studies including both men and women (Sjölund et al., 2015). The latter study, moreover, found no effect modification by sex, indicating that the influence of intellectual ability on alcohol‐related disorders is comparable in men and women. We found that baseline IQ scores were not only associated with alcohol‐related hospital diagnoses but also with self‐reported treatment for alcohol problems, which is in accordance with a previous longitudinal study observing an association between low IQ scores and self‐reported risky drinking (Mortensen et al., 2006). Although only the crude associations were presented because the analyses of baseline intelligence test scores and alcohol‐related disorders were merely related to the secondary aim, it deserves mentioning that the associations remained statistically significant after adjustment for both year of birth and school education. Thus, IQ scores seem to be associated with alcohol‐related disorders and not just with the risk of hospitalization or the likelihood of getting the alcohol‐related diagnosis.

### Alcohol‐Related Disorders and Changes in Intelligence Test Scores

Our findings of additional decline in intelligence test scores observed in individuals with alcohol‐related disorders are less well‐supported as longitudinal studies including intelligence assessed both before and after disease initiation are lacking. In general, few studies have been able to identify factors influencing individual differences in age‐related intellectual decline (Plassman et al., 2010). In this study, we identified a modifiable lifestyle factor that explained around 2% of the variance in changes in IQ scores. Moderate explained variance is expected given the numerous potential factors influencing lifelong intellectual changes. Nevertheless, alcohol‐related disorders were associated with a decline in IQ test scores between −4 IQ points (self‐reported treatment for alcohol problems) and −5 IQ points (alcohol‐related hospital diagnoses), corresponding to between ¼ and ⅓ of the standard deviation of 15, which underlines the importance of our results.

In a cross‐sectional study, Schottenbauer and colleagues (2007) investigated whether the association between alcohol dependence and intellectual performance differed by participants’ age. Results of these analyses indicated that higher vocabulary was observed with higher age regardless of alcohol dependence status; whereas performance on block design was lower with higher age in individuals with alcohol dependence but not in controls. Hence, alcohol dependence appeared associated with age‐related decline in fluid intelligence but not in verbal intelligence (if age cohort differences in intelligence and alcohol dependence are assumed not to distort the findings). Although we could not investigate changes within specific domains of intelligence in the present study, the cross‐sectional results of Schottenbauer and colleagues can be interpreted as consistent with our results of more decline in IQ scores in individuals with alcohol‐related disorders because the BPP subtests require reasoning and may primarily assess fluid intelligence (Hartmann and Teasdale, 2004, 2005). Moreover, our finding of larger decline in IQ scores in individuals with somatic alcohol‐related hospital diagnoses may indicate that individuals with more severe and long‐lasting alcohol‐related disorders are particularly at risk of intellectual decline. Similarly, analyses of number of alcohol‐related hospital registrations in the 207 men with alcohol‐related hospital diagnoses suggest a dose–response association in terms of larger decline in IQ scores with increasing number of registrations (estimated IQ decline per additional registration = −0.15; *p* = 0.008). These results are in accordance with a cross‐sectional study by Glass and colleagues (2009) suggesting a negative effect of severity of alcoholism on intellectual functioning.

It has important implications for preventive strategies whether the observed associations between alcohol‐related disorders and changes in intelligence test scores are a result of direct influences of alcohol on the brain or indirect influences of, for example, comorbidity. To assess whether the associations were mainly driven by direct effects of alcohol on intellectual performance, we conducted analyses adjusting the associations for average weekly units of alcohol consumed in adult life and years with weekly extreme binge drinking (≥10 units of alcohol on the same occasion) in adult life using information that was retrospectively self‐reported at the follow‐up examination. The estimates of decline in intelligence test scores with alcohol‐related disorders were attenuated, but alcohol consumption did not appear to entirely explain the observed associations. Although this may partly be due to misclassification of self‐reported alcohol consumption, it is also likely that factors other than amount of alcohol consumed influence the associations. Alcohol‐related disorders may, for example, be associated with adverse psychological, physical, and social factors that are unrelated to alcohol consumption but related to intellectual decline. In the analyses including adjustment for psychiatric and somatic disease, the estimates of changes in intelligence test scores with alcohol‐related disorders were likewise attenuated, indicating that some—but probably not all—of the association may be explained by comorbidity. However, residual confounding by comorbidity is probably inevitable even though it was obtained using register information from Danish hospitals. For example, diseases not requiring hospital admissions would not be included.

Previous clinical neuropsychological studies and MRI studies have indicated that at least a part of the structural brain changes and intellectual changes associated with alcohol‐related disorders diminish with periods of alcohol abstinence (Stavro et al., 2013; Sullivan, 2017). More specifically, the studies included in the meta‐analysis by Stavro and colleagues observed only small differences in intelligence test scores between normal controls and individuals with alcoholism who had been long‐term abstinent for at least a year (2013). Our analyses excluding individuals with alcohol‐related diagnoses within a year before the follow‐up test session and our analyses including only currently abstinent cases suggested that only a small part of the association between alcohol‐related disorders and changes in intelligence test scores was explained by acute effects of alcohol on intellectual performance. As it can be argued that alcohol abstinence is not the only successful treatment outcome, analyses were also conducted including adjustment for heavy alcohol consumption the past year, suggesting highly similar results. Thus, significantly larger decline in intelligence test scores in individuals with alcohol‐related disorders compared with individuals without these disorders was still present, which may indicate that the changes in intellectual performance with alcohol‐related disorders are not only acute. Nevertheless, more studies are needed to confirm the presence of permanent intellectual decline with alcohol‐related disorders.

### Strengths and Limitations

The main strength of this large‐scale study is the quality of the included variables. First, alcohol‐related disorders were assessed in 2 different ways, enabling us to investigate whether these 2 assessment methods—affected by different limitations—revealed similar results. Second, intelligence was assessed using the same intelligence test in early adulthood and late midlife, that is, before and after onset of alcohol‐related disorders. Therefore, it was possible to examine the influence of intelligence test scores on alcohol‐related disorders in addition to the influence of these disorders on changes in intelligence test scores. Third, it is a major strength that the study included several important covariates, comprising detailed measures of alcohol consumption, pack‐years of smoking, as well as psychiatric and somatic comorbidity.

Misclassification of intellectual changes may have occurred although the same intelligence test was administered to the participants in young adulthood and late midlife. First, the change from paper‐and‐pencil to computer‐based completion may per se have resulted in changes in total intelligence test scores. Second, some young men may have performed less well on the test in young adulthood to avoid military service, resulting in an underestimation of the intellectual decline. It could, however, be argued that the amount of both types of misclassification of intellectual changes is probably unrelated to the alcohol‐related disorder status of participants; hence, the estimated group differences are not necessarily influenced.

The 2 assessment methods of alcohol‐related disorders—hospital diagnoses and self‐reported treatment—both have limitations that may cause misclassification. Hospital diagnoses only include individuals treated at hospital departments, whereas treatment for alcohol problems was selectively reported by participants and does not include specific diagnoses. As participants were born in 1950 to 1961 and the Danish National Patient Registry and the Danish Psychiatric Central Research Register were not initiated until 1977 and 1969, respectively, misclassification of individuals with early alcohol‐related disorders may be present. However, these individuals are presumably captured by the self‐reported treatment for alcohol problems. The strikingly similar results observed using the 2 different assessments indicate that both assessment methods capture individuals with alcohol‐related disorders. Moreover, as the self‐reported treatment for alcohol problems also includes individuals getting treatment not at hospitals, the similar results may indicate that the intellectual changes related to alcohol‐related disorders are not fundamentally different among individuals seeking help at different sites. In addition to misclassification, early heavy alcohol consumption and unregistered alcohol‐related disorders may introduce problems of reverse causation. Individuals with early heavy alcohol consumption and alcohol‐related disorders are at higher risk of alcohol‐related disorders later in life, and the intelligence test score in young adulthood is probably lower due to the concurrent heavy alcohol consumption. Thus, reverse causation may explain some of the observed association of low baseline intelligence test scores with alcohol‐related disorders. In contrast, the decline in intelligence test scores related to alcohol‐related disorders would presumably be underestimated due to this reverse causation, assuming that low baseline intelligence test scores will lead to less decline if the follow‐up test score is not influenced by concurrent heavy alcohol consumption.

The selection of participants is a core concern—like in previous studies using data from the Lifestyle and Cognition Follow‐up study 2015 (LiKO‐15)—due to the potentially limited generalizability and the risk of selection bias. Selection of participants took place first at the draft board examination and second at the follow‐up test sessions. At draft board examination, women were not required to appear and men with certified disqualifying diseases (5 to 10%) were exempted. At follow‐up test sessions, only men living within 50 km from test locations who were draft board examined in greater Copenhagen or the island of Bornholm were invited. Moreover, register information on psychiatric hospital admissions was used to invite a larger proportion of participants with psychiatric disorders. The most worrying selection is, nevertheless, the self‐selection at follow‐up, that is, whether the invited men agreed to participate in the follow‐up test sessions. In addition to the previously suggested higher education, higher intelligence test scores, and lower morbidity in LiKO‐15 participants than nonparticipants (Grønkjær et al., 2019b), men with alcohol‐related hospital diagnoses were less likely to participate in the follow‐up study than men without these diagnoses. Although selection bias due to self‐selection cannot be ruled out, sufficient variance in all included factors was still apparent among participants.

In this large male study sample, we observed lower intelligence test scores in young adulthood in individuals with alcohol‐related disorders regardless of whether these disorders were assessed using hospital diagnoses or self‐reported treatment for alcohol problems. In addition, men with alcohol‐related disorders had a larger decline in intelligence test scores from young adulthood to late midlife than men without these disorders. Thus, lower intellectual ability in individuals with alcohol‐related disorders observed in clinical studies is probably a result of both lower premorbid intelligence and more intellectual decline in this group compared with individuals without alcohol‐related disorders.

## Funding

The Lifestyle and Cognition Follow‐up study 2015 is part of the Phenotypes in Alcohol Use Disorders project, which is supported by Innovation Fund Denmark, Health and Clinical Research (grant number 603‐00520B). Marie Grønkjær is supported by a PhD Scholarship grant from the Faculty of Health and Medical Sciences, University of Copenhagen.

## Conflicts of Interest

The authors have no conflicts of interest to declare.

## Supporting information


**Table S1**. Psychiatric and somatic alcohol‐related hospital diagnoses in the study sample of 2,499 Danish men according to International Classification of Disease (ICD) codes.

**Table S2.** Adjusted estimates of changes in IQ scores with *somatic* alcohol‐related hospital diagnoses in 2,499 Danish men.
