# Supplementary material for: Intelligence Test Scores Before and After Alcohol‐Related Disorders—A Longitudinal Study of Danish Male Conscripts
Source: Alcohol Clin Exp Res. 2019 Aug 24;43(10):2187–95. doi: 10.1111/acer.14174 (PMC6851852; doi:10.1111/acer.14174)
Supplement: Supplementary file 1 — Table S1. Psychiatric and somatic alcohol‐related hospital diagnoses in the study sample of 2,499 Danish men according to International Classification of Disease (ICD) codes. [file ACER-43-2187-s001.docx]

Table S1 Psychiatric and somatic alcohol-related hospital diagnoses in the study sample of 2,499 Danish men according to International Classification of Disease (ICD) codes

| *Diagnoses* | *ICD code* | *N* |
| --- | --- | --- |
| *Psychiatric alcohol-related hospital diagnoses* |  |  |
| **ICD-8 codes** |  |  |
| Delirium tremens | 29109 | 4 |
| Psychosis korsakov(alcoholica) | 29119 | 0 |
| Hallucinosis alcoholica alia | 29129 | 0 |
| Paranoia alcoholica | 29139 | 0 |
| Psychosis alcoholica alia et non specificata | 29199 | 0 |
| Alcoholismus episodicus | 30309 | 17 |
| Alcoholismus habitualis | 30319 | 16 |
| Alcoholismus chronicus | 30320 | 9 |
| Alcoholismus chronicus alius definitus | 30328 | 0 |
| Alcoholismus addictivus | 30329 | 23 |
| Polyneuritis alcoholica | 30391 | 0 |
| Alcoholismus alia et non specificata | 30399 | 10 |
| ICD-10 codes |  |  |
| Harmful use | F10.1 | 56 |
| Dependence syndrome | F10.2 | 142 |
| Withdrawal state | F10.3 | 59 |
| Withdrawal state with delirium | F10.4 | 12 |
| Psychotic disorder | F10.5 | 7 |
| Amnesic syndrome | F10.6 | 1 |
| Residual and late-onset psychotic disorder | F10.7 | 5 |
| Other mental and behavioural disorders | F10.8 | 1 |
| Unspecified mental and behavioural disorder | F10.9 | 4 |
| Total number of psychiatric alcohol-related hospital diagnoses |  | 366^*^ |
| (ICD-8 and ICD-10) |  |  |
| *Somatic alcohol-related hospital diagnoses* |  |  |
| **ICD-8 codes** |  |  |
| Cirrhosis hepatis ex alcoholismo(laennec) | 57109 | 1 |
| Steatosis hepatis alcoholica | 57110 | 1 |
| ICD-10 codes |  |  |
| Alcohol-induced pseudo-Crushing syndrome | E24.4 | 0 |
| Degeneration of nervous system due to alcohol | G31.2 | 1 |
| Alcoholic polyneuropathy | G62.1 | 3 |
| Alcoholic myopathy | G72.1 | 0 |
| Alcoholic cardiomyopathy | I42.6 | 0 |
| Alcoholic gastritis | K29.2 | 7 |
| Alcoholic liver disease | K70 | 26 |
| Alcohol-induced acute pancreatitis | K85.2 | 1 |
| Alcohol-induced chronic pancreatitis | K86.0 | 9 |
| Total number of somatic alcohol-related hospital diagnoses |  | 49^*^ |
| (ICD-8 and ICD-10) |  |  |

^*^As some individuals have been registered with more than one alcohol-related hospital diagnosis, the total number of diagnoses adds up to more than the total number of individuals with alcohol-related hospital diagnoses presented in Table 1–4.
