# Supplementary material for: Intelligence Test Scores Before and After Alcohol‐Related Disorders—A Longitudinal Study of Danish Male Conscripts
Source: Alcohol Clin Exp Res. 2019 Aug 24;43(10):2187–95. doi: 10.1111/acer.14174 (PMC6851852; doi:10.1111/acer.14174)
Supplement: Supplementary file 2 — Table S2. Adjusted estimates of changes in IQ scores with somatic alcohol‐related hospital diagnoses in 2,499 Danish men. [file ACER-43-2187-s002.docx]

**Table S2** Adjusted estimates of changes in IQ scores with *somatic* alcohol-related hospital diagnoses in 2,499 Danish men

|  | *Mean group difference  in IQ changes (95% CI)* | *Increment in R^2^ by the  alcohol-related disorder variable* | *P-value* |
| --- | --- | --- | --- |
| *Somatic* alcohol-related hospital diagnoses (N_cases_=40) |  |  |  |
| Unadjusted estimates | -7.1 (-10.0;-4.2) | 0.9% | <0.001 |
| Model 1-adjusted estimates | -7.6 (-10.2;-4.9) | 1.0% | <0.001 |
| Model 1 + psychiatric and somatic comorbidity | -5.6 (-8.3;-2.9) | 0.5% | <0.001 |
| Model 1 + average units of alcohol/week in adult life | -7.1 (-9.8;-4.4) | 0.9% | <0.001 |
| Model 1 + years with weekly extreme binge drinking in adult life | -6.4 (-9.1;-3.7) | 0.7% | <0.001 |

IQ = intelligence quotient calculated using Børge Priens Prøve test scores, Model 1: adjusted for year of birth, retest interval length, baseline IQ score, years of education and pack-years of smoking.

One unit of alcohol = 12 grams of pure alcohol. Extreme binge drinking defined as consuming 10 units of alcohol or more on the same occasion.
